# Surveillance of *Clostridium difficile* infections in a long-term care psychogeriatric facility: outbreak analysis and policy improvement

**DOI:** 10.1186/s13690-015-0067-y

**Published:** 2015-04-06

**Authors:** Gretel Van Esch, Johan Van Broeck, Michel Delmée, Boudewijn Catry

**Affiliations:** Scheutbos, Revalidation Centre for Psychogeriatric patients, Brussels, Belgium; Department of Clinical Microbiology, Cliniques Universitaires Saint-Luc (UCL), Brussels, Belgium; Healthcare-associated Infections (NSIH) & Antimicrobial Resistance, OD Public Health & Surveillance, Scientific Institute of Public Health, Brussels (WIV-ISP), Rue Juliette Wytsmanstraat 14, 1050 Brussels, Belgium

**Keywords:** *Clostridium difficile*, Intervention, Nutritional status, LTCF

## Abstract

**Background:**

Following an exceptionally high *Clostridium difficile* infections (CDI) incidence (Spring 2011) in a psychogeriatric long-term care facility, a bidirectional study (2009–2012) was initiated to identify determinants (retrospectively) and to assess intervention measures taken (prospectively).

**Methods:**

For every CDI patient (*de novo* cases, relapses, and recurrences), a control patient (patient in the opposite room) was selected and risk factor analysis performed. Following the epidemic peak a more stringent hygienic protocol and surveillance program were implemented, as well as uniform guidelines for metronidazole and vancomycin prescription.

**Results:**

The nutritional state (total protein/prealbumine) significantly differed between the CDI group (poorer nutritional state at admission) and the control group, and also antibiotic use (general) could be confirmed as a risk factor. A multi-disciplinary nutritional team has been established in order to improve the nutritional balance of our patients.

**Conclusions:**

Aside from stringent hygiene and antibiotic prescription stewardship, malnutrition of patients is a factor to be taken into account to contain a CDI outbreak in a long term care facility (LTCF).

## Background

*Clostridium difficile* is an anaerobic Gram-positive, spore-forming bacterium [1]. The ability of *C. difficile* to form spores is thought to be a key feature in enabling the bacteria to persist in patients and in the physical environment for long periods, thereby facilitating its transmission. *C. difficile* is transmitted through the fecal-oral route. The risk factors for acquiring *C. difficile* include contact with a contaminated environment, contact with persons who are infected with and shedding *C. difficile*, and ingestion of contaminated food [2].

As the many spores formed by *C. difficile* are resistant to most routine cleaning methods used on surfaces in hospitals (except for diluted bleach), *C. difficile* infection (CDI) has become the leading cause of diarrhea and pseudomembranous colitis in healthcare settings [3]. There is a strong connection between antimicrobial therapy and CDI, as *C. difficile* can only colonize the gut if the normal intestinal flora is disturbed or absent [3]. In recent years, an increase in the incidence and the severity of healthcare-associated CDI (nosocomial) has been reported worldwide. This had been attributed to multiple factors including a changing demographic situation, increased use of broad-spectrum antibiotics and the emergence of hypervirulent *C. difficile* strains. Outbreaks were associated with the emergence and rapid spread of a specific strain of *C. difficile* belonging to the PCR-ribotype 027 or pulsotype NAP1 (North American Pulsotype 1) [3]. Some of the characteristics of this strain are higher in vitro production of toxins A and B and presence of a third toxin named binary toxin. Since about 2005, the epidemic strain has started to circulate in northern Europe (United Kingdom, Belgium and the Netherlands) [1].

Older people are particularly susceptible and at increased risk for adverse outcomes as a result of CDI. The increased risk of acquiring CDI in the elderly may be due to age-related changes in intestinal flora, immune senescence, or the presence of other underlying diseases [3].

In Belgium, CDI incidence more than doubled between 1998 and 2007, after which it stabilized, be it at a high level [4,5]. After an initial increase, the CDI mortality has been decreasing since 2004 [4]. PCR ribotype 027 was identified for the first time in 2003. Although strains attributed to severe pathology are generally found in hospital patients, recent reports suggest that the occurrence and severity of CDI in the community is also increasing. Moreover, CDI is more and more recognized as a cause of diarrhea in populations previously considered to be at low risk. The carriage ratio of *C. difficile* in the community-dwelling elderly population in the UK is 4% [6]. The patient population in the psychogeriatric institutions is marked by cognitive impairment which hampers an optimal maintenance of standards of personal hygiene. Patients are often unconscious of their hygienic condition and nursing staff regularly has to deal with non-cooperative behavior regarding personal hygiene.

The long-term psychogeriatric facility, from which the data in this manuscript are observed, had an increase of CDI between 2009–2010 and 2012. However in the beginning of 2011, a substantial increase of CDI episodes was recorded in the institute. The objective of the manuscript was to investigate underlying causes of this epidemic episode in order to identify the risk factors within this specific population and health care facility.

## Methods

### Study designs

#### A. Retrospective design

To identify risk factors for CDI, data covering the period of January 2009 to December 2012 were retrospectively abstracted from the patient’s record in four departments of one psychogeriatric facility. The hospital had a total capacity of 120 beds and the mean length of stay was 34 days. The effect was evaluated statistically.

#### B. Prospective design

To improve the CDI policy within institutions, an active surveillance and prevention campaign (= intervention) was launched during the epidemic peak in Spring 2011, including hygiene precautions for health care professionals and visitors. The effect was evaluated descriptively until the end of the study period, namely December 2012.

### Intervention

A more stringent hygienic protocol and surveillance program were implemented in order to better battle the current outbreak and to sooner detect potential future outbreaks: All CDI occurrences were reported back to a single reference person and analyzed on a three-monthly basis. When an increase by more than two standard deviations above the normal background level was observed, the following measures were taken: (a) staff was alerted of the increased CDI incidence and the importance of the existing hygienic measures was explicitly impressed upon all personnel and visitors; (b) strict isolation measures were immediately taken for patients with diarrhea awaiting the laboratory results on their fecal samples; (c) patients with high suspicion for CDI (characteristic odor and mucal composition of diarrhea) were immediately treated with metronidazole (p.o. 3 × 500 mg daily for 10 days) or vancomycin (p.o. 4 × 125 mg daily for 10 days); (d) patients with confirmed CDI must have been treated with vancomycin and were kept in isolation during the full course of their CDI treatment (10 days for *de novo* cases, 21 for relapses); (e) at the end of any CDI treatment, patient rooms were cleaned with diluted bleach (NaClO) prior to the standard disinfection procedure. This cleaning procedure was once more repeated when the concerned patient was discharged.

A request for ribotyping was submitted to the local ethical committee (Number of acceptance: 2012/03/09/2) in order to assess which strain was circulating within the institution.

Upon approval (29 May 2012), a ribotyping was done among all consecutive patients within the span of one year (10 patients in total) in the aftermath of the 2011 peak.

### Case definition & bacteriology

A *Clostridium difficile* infection (CDI) was defined if a patient showed diarrhea as the major clinical symptom and had a positive toxin/antigen test (C. diff quik chek complete® - Alere Techlab) and a positive stool culture.

Cases were classified as (I) *de novo* case: no prior infection known; (II) relapse: CDI within 2 weeks after completing the treatment, and (III) late relapse (or recurrence): CDI later than 2 weeks after completing the treatment.

*C. difficile* culture was performed according to local standard procedures (CPG laboratory Brussels) (selective media: CLO medium, incubation at 35° for 48 hours under anaerobic conditions). Colonies were identified based on Gram-staining, typical odor and chartreuse fluorescence under ultraviolet light.

*C. difficile* isolates (only from the peak of 2011 onwards, i.e. after initiation of intervention), were sent to the national reference laboratory [7] (UCL Brussels) for typing. Strains were characterized by PCR-ribotyping, based on the comparison of patterns of PCR products of the 16S–23S rRNA intergenic spacer regions using primers described by Barbut *et al.* [8]. The size of each peak was determined using GeneScan software or GeneMapper V.4 software (AB - Applied Biosystems) [5].

### Patient data recovery and analysis

For all CDI episodes, patients’ files were reviewed (G VE). For each patient, the patient in the opposite room served as control. The parameters that were extracted are presented in Table 1.

**Table 1:** **Overview of patient characteristics abstracted from the medical records of** ***Clostridium difficile*** **cases and controls in a long-term psychogeriatric health care institute, Belgium 2011**

General patient characteristics:	
**Age**	(years) at admission
**Body Mass Index (BMI)**	(kg/m^2^) at admission
**Frailty index (FI)**	at admission and at discharge according to Drubbel *et al.* [9] (scaling between 1 and 0, 0 being perfectly healthy)
**Nutritional status**	(mg/dL or g/L) at admission based on prealbumine: (<20 mg/dl = malnutrition, >20 mg/dL = normal). If not available, based on total protein: <5,0 g/L malnutrition, >5,0 g/L normal) [10]
**Mini Mental State Examination (MMSE)**	(x/30) during stay to assess the cognitive functioning. MMSE below 21/30 is a usual cut-off to define dementia [11]
CDI specific characteristics:	
**Interval between admission and infection**	(days), the date at which lab results confirm the clinical diagnosis is taken as the date of infection
**Length of stay**	(days) between admission and discharge
**Type of treatment for CDI**	metronidazole or vancomycin
**Length of CDI treatment**	(days)
**Mortality**	(%)
Literature reported CDI risk factors:	
**Type of residency**	one month prior to admission, i.e. previous health care exposure (acute hospital, nursing home, LTCF)
**Number and type of antibiotics**	used one month prior to CDI
**Antacids**	used at admission (proton pump inhibitor or histamin-2 blockers or none)
**Number of narcotics**	used at admission
**Diabetes mellitus type 2**	at admission (yes/no)

CDI: *Clostridium difficile* infection; LTCF: long-term care facility.

The statistical analysis of the patient parameters was performed based on the CDI incidence. This approach inherently applied the appropriate weight factor to the patient parameters of the more susceptible patients, as the data for a patient who had two infections were counted twice. All statistical analysis were performed either with Microsoft Excel 2007 or with SPSS Statistics version 17.0. (SPSS Inc, Chicago, IL). The *t*-test for independence and Chi squared-test for nominal data were applied, with significance level set at 0,05.

## Results

### A. Retrospective patient study

The study population consisted of 66 patients affected by *C. difficile* and 61 control patients. A total of 94 episodes were included in the study: 42 patients had one single episode of CDI during their hospitalization in the institution and 24 patients relapsed or had a late relapse. Among the latter, 20 patients had 2 episodes (16 relapses and 4 late relapses), 3 patients with 3 episodes (4 relapses and 2 late relapses), and 1 patient with 4 episodes of CDI (3 relapses and 0 late relapses), respectively.

**Figure 1:**
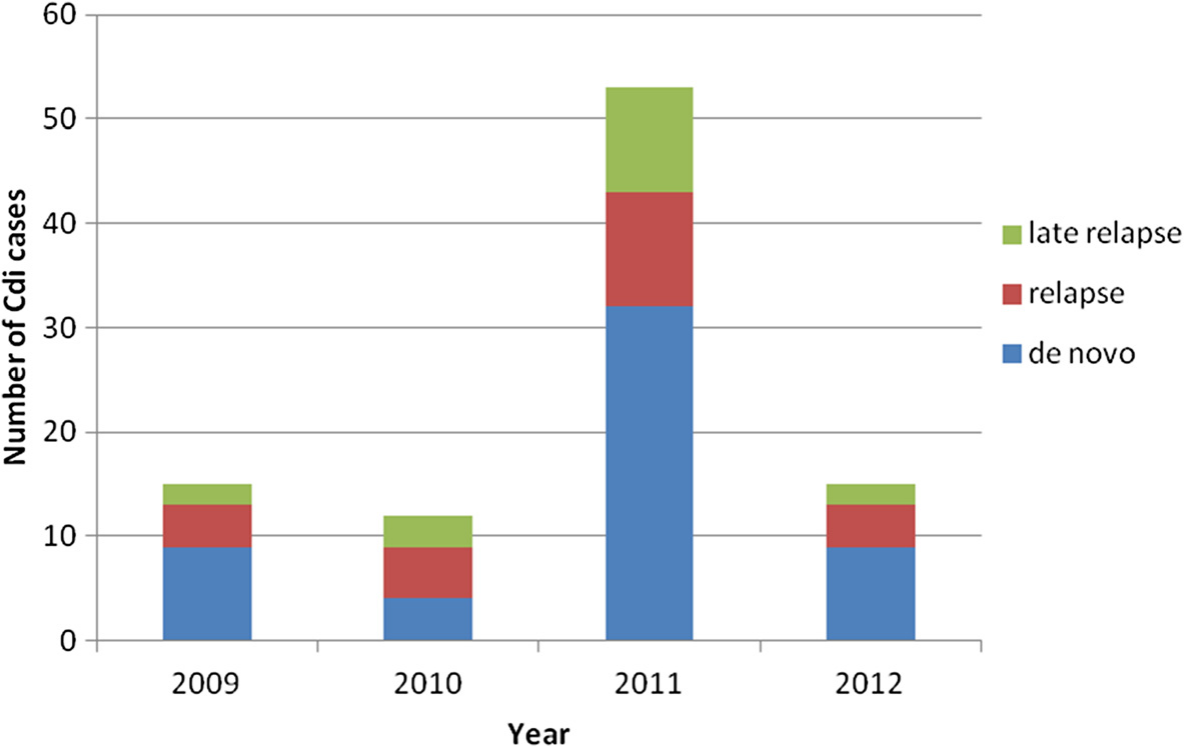
**Graphical overview of the incidence of** ***Clostridium difficile*** **infection (CDI) in a long-term psychogeriatric institution between 2009 and 2012, Belgium.**

A baseline CDI incidence of 16 cases/1000 admissions was found for the years 2009, 2010, and 2012, respectively. Figure 1 shows the epidemic increase in the number of CDI cases in 2011. The increase included the total number of CDI episodes, i.e. *de novo* cases, relapses and late relapses.

The institution underwent an increased CDI incidence as also reported by others during the same time frame in Europe [12]. Once introduced, the highly infectious strain spreads in-house: 42% of the relapses occurred for patients in rooms previously occupied by an infected patient or in adjacent rooms.

#### General patient characteristics

Table 2 gives the association comparing patients’ characteristics of any CDI events and controls. The analysis accounted for the fact that patients with a relapse would have been counted more than once. Patients in the infected group showed near-identical general characteristics compared to patients in the control group. No difference was found regarding their mean age, body mass index (BMI), frailty index or MMSE (Mini Mental State Examination).

**Table 2:** **Comparison of patients’ characteristics of** ***Clostridium difficile*** **infection (CDI) events and controls**

	**Controls**			**CDI infections**			**p-value** ^**§**^
	**Mean**	**St. Dev.**	**Range**	**Mean**	**St. Dev.**	**Range**	
Age (years)	82	7,3	(64–99)	82	7	(66–97)	0,77
BMI (kg/m^2^)	23	5,1	(15–37)	23	5,5	(15–40)	0,88
FI-admission	0,38	0,09	(0,25-0,55)	0,38	0,07	(0,22-0,53)	0,62
total protein (g/L)	5,8	0,9	(4–8)	5,4	0,8	(4–8)	0,03*
Prealbumine (mg/dL)	20,6	7	(8–35)	16,6	7,4	(5–41)	0,03*
MMSE (x/30)	22	6	(7–30)	21	7,3	(0–30)	0,68

^§^p-value: from independent *t*-test; *: significant at p<0,05.

For abbreviations: see Table 1.

#### CDI specific characteristics

As discussed in the above section, especially during the 2011 CDI incidence peak, the institute had an important influx of patients carrying the CDI. Of all positive cultures, 18% were obtained within one week after admission and up to 25% within two weeks. We can assume that the majority of the former had contracted *C. difficile* before admission in our hospital.

The mean length of stay for the control group was 55 days and for the CDI patient group 85 days. Logically, the infection prolonged their mean length of stay, partially due to the length of the CDI treatment itself, and partially due to their weakened state thereafter requiring additional care.

**Figure 2:**
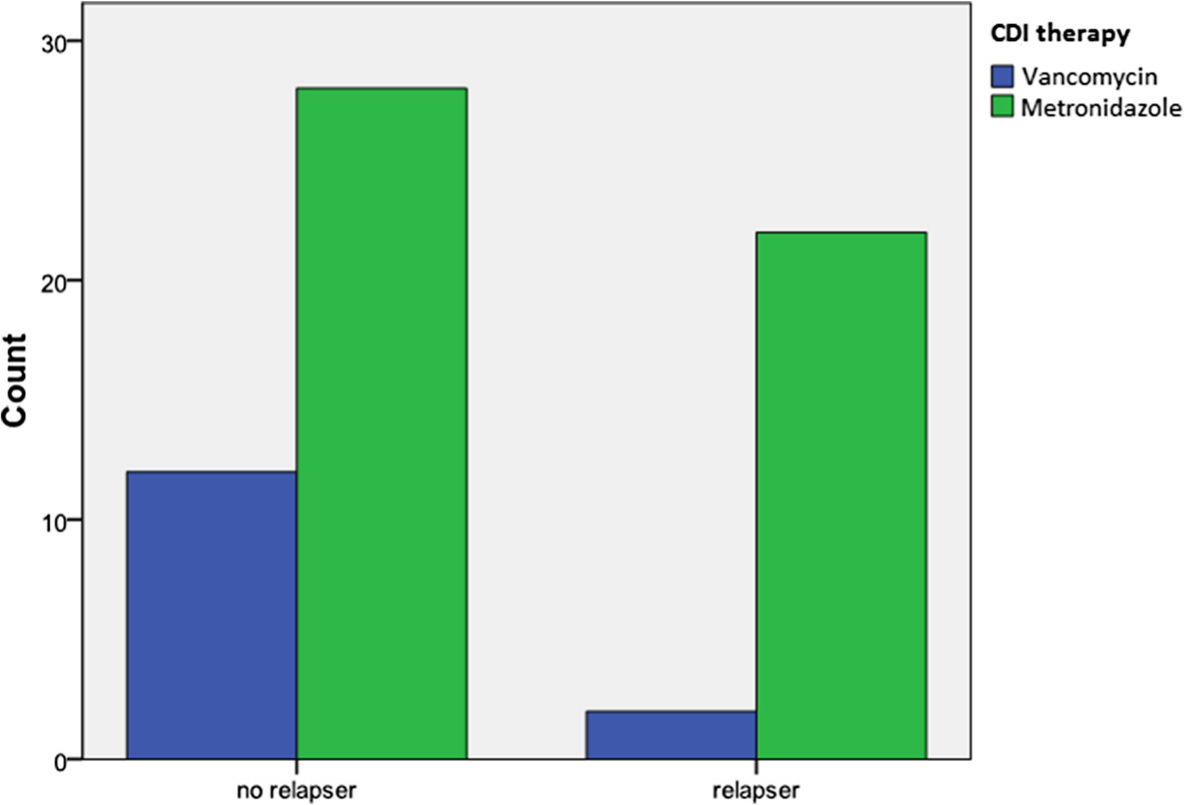
**Vancomycin (blue) or metronidazole (green) treatment counts for** ***Clostridium difficile*** **infections (CDI), separated between relapse and non-relapse cases.**

Figure 2 presents the antimicrobial treatment in function of case classification. The odds of relapse were 4,3 times (95% CI: 0,867 – 21,550) higher in patients treated with metronidazole treatment compared to vancomycin treatment. Metronidazole reduced sensitivity is known for the ribotype 027 strain [13]. Although no ribotyping was performed at the moment of the outbreak, this finding suggested that we dealt with the same strain as the one previously identified in the acute hospital setting. The odds for relapse when comparing a short versus longer treatment, i.e. less than 14 days or more than 14 days, showed no benefit for the longer treatment in our study population (odds ratio = 0,9 (95% CI: 0,300 – 2,502)).

The mortality in the CDI group was 21% compared to 10% in the control group.

#### Literature reported CDI risk factors

Since literature reported CDI risk factors, as selected for this study, have all been evaluated at admission, we have chosen to perform this statistical analysis based on the data per patient rather than per CDI incidence event. An overview of the statistical analysis is shown in Table 3. From the risk factors previously discussed in literature, only antibiotic use differs significantly between the control group and the CDI patients in our study population.

**Table 3:** **Statistical analysis of the literature reported** ***Clostridium difficile*** **infection (CDI) risk factors for all patients in the study**

	**Controls**		**CDI patients**		**p-value**
	**No.**	**%**	**No.**	**%**	
Previous health care exposure	53/61	87	64/66	97	0,152
• Acute hospital	52/53	98	62/64	97	
• Nursing home	1/53	2	1/64	1,5	
• LTCF^a^	0/53	0	1/64	1,5	
Any antibiotic use during 1 month preceding symptom onset	33/52	63	58/66	88	0,001*
• Clindamycin	1/49	2	2/84	2,3	
• Fluoroquinolones	7/49	14	13/84	15,4	
• Amoxicillin–clavulanic acid	22/49	45	36/84	43	
• Other^b^	19/49	39	33/84	39	
Antacids use at admission	39/55	71	48/57	84	0,138
• Histamine 2-blocker	5/39	13	3/48	6	
• Proton-pump inhibitor (PPI)	34/39	87	45/48	94	
Number of narcotics at admission	47/53	89	48/58	83	0,832
• 1 narcotic	21/47	45	17/48	35	
• 2 narcotics	15/47	32	16/48	33	
• 3 narcotics	7/47	15	11/48	23	
• 4 narcotics	3/47	6	3/48	6	
• 5 narcotics	1/47	2	1/48	2	
Diabetes mellitus	14/57	25	11/64	17	0,317

^a^LTCF: long-term care facility.

^b^’other’ includes cephalosporins, tetracyclines, macrolides and cotrimoxazole (trimethoprim + sulphonamides).

The last column reports the p-value of Chi^2^-test for nominal data. The values marked with the asterix are significant at the 0,05 level.

### B. Prospective CDI policy improvement

**Figure 3:**
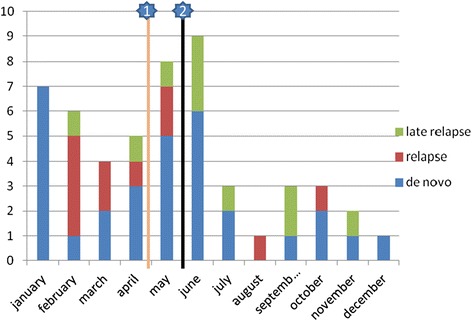
**Graphical overview of the monthly incidence of** ***Clostridium difficile*** **infection (CDI) in the institution in 2011.** Line 1: First awareness moment with communication of increase in incidence. Line 2: Formal intervention start, with stringent hygienic protocol, surveillance and uniform antibiotic guidelines.

Although hygienic procedures were available at the moment of the outbreak, raising awareness and improved internal communication were the first steps to be undertaken. Figure 3 shows that the implementation of the infection control measures (May-June 2011) coincided with a significant drop in CDI incidence. Although the epidemiological characteristics combined with the metronidazole reduced sensitivity strongly point towards infections with the 027 strain, ribotyping was not able to confirm this suspicion, given the time delay between the outbreak and the approval from the ethical committee.

Upon approval of the request (May 2012), all consecutive patients (n = 10) within the time frame of one year, were ribotyped, but no 027 strain was confirmed in any of these samples. Reassuringly, these findings substantiate the effectiveness of the more stringent hygienic protocol for battling an infectious outbreak.

The patient parameter study has led to two additional measures; (a) guidelines for metronidazole and vancomycin prescription have now been implemented as described above, and (b) if malnutrition appears to be an underlying problem and a possible additional risk-factor, a multi-disciplinary nutritional team, consisting of a psychologist, dietician and physiotherapist is established in order to improve the nutritional balance of the patients.

## Discussion

Only the nutritional status, based on the total protein and prealbumine levels, was found to be significantly poorer for the patients that suffered CDI: whereas patients in the control group on average were classified as normal (mean prealbumine >20 mg/dL), patients in the infected group already suffered from malnutrition at admission (mean prealbumine < 20 mg/dL). Their total protein level was also significantly lower. A multi-disciplinary nutritional team thus can be considered in long term care facilities to further contain *C. difficile* outbreaks.

Within the general patient characteristics, the frailty index was higher than reported by Drubbel *et al.* [9]. They reported a median FI score of 0,08 for women and 0,06 for men. Our median FI was 0,39. A slight discrepancy could arise from the fact that we calculated the FI manually, based on the patients files and not automatically as done by Drubbel and her group. However, the main difference is related to the patient population itself: in contrast to their study population (community-dwelling elderly people), here we only focused on inpatients.

To our knowledge, there is only one study evaluating cognitive impairment in relation with CDI. Kyne *et al.* [14] reported in 1999 that cognitive impairment is a risk factor for severity of CDI. Although here, we also hypothesized that cognitive impairment might be an additional risk factor for contracting or developing CDI, our data did not support this hypothesis: no difference was observed between the MMSE data of both groups. Given the high frailty index (FI) in the population of our study, it should be noted that both groups are dependent on the same external help for their hygienic situation, independent of their cognitive state. Thirty-eight percent of our *C. difficile* patients and 36% of our control patients went to a nursing home after discharge.

Regarding the CDI specific parameters, we noticed an overall mortality rate of 21% in our CDI group. This number is in the range of other mortality rates published before [15,16]. As we did not calculate the attributable mortality of CDI, we probably overestimate the CDI mortality rate because we did not correct for other factors like high age and multiple underlying diseases [17].

The increasing incidence and severity of CDI is largely described in literature [12]. Viseur *et al.* published the surveillance data for Belgium from 2008 to 2010 [5]. Data from these years showed a relatively stable incidence of CDI in Belgian hospitals, with a slight decrease in 2010. Surveillance data until 2011 are available on the website of the Public Health and Surveillance Department, Scientific Institute for Public Health (WIV-ISP), Brussels [4]. The overall incidence of CDI in 2011 is comparable with 2010, but between 2007 and 2011, there were more outbreaks of CDI in long-term care hospitals than in the acute hospital setting (60% vs. 38%). Our data fits within this trend. The background incidence (16 CDI per 1000 admission) is comparable to the data reported by Mascart *et al.* in a geriatric department (Centre Hospitalier Universitaire Brugmann, Brussels), located in the same geographic area [15]. They observed an incidence of 13/1000 admissions in 2008 and 21/1000 admissions in 2009. The ribotype 027 was the most prevalent ribotype during the years 2007–2009. Unfortunately no recent data are available to support the hypothesis of the ribotype 027 involvement in outbreaks among patients in geriatric wards.

Based upon experience, vancomycin could be recommended as the first-line agent to protect against recurrence and control of a hospital outbreak caused by a hypervirulent strain. Control is difficult but possible through a combination of barrier precautions, environmental cleaning, and antimicrobial stewardship, interventions that all are concordant with literature [13,15].

## Conclusions

In summary, following the 2011 CDI outbreak in the institution in which the study was conducted a more stringent hygienic protocol and surveillance program were implemented in order to sooner detect potential outbreaks. Additionally, uniform guidelines for metronidazole and vancomycin prescription have been implemented. Finally, a multi-disciplinary nutritional team has been established in order to improve the nutritional balance of our patients.

### Ethical standards

The authors assert that all procedures contributing to this work comply with the ethical standards of the relevant national and institutional committees on human experimentation and with the Helsinki Declaration of 1975, as revised in 2008.
